# Interpretation of results of PCR and B-D-glucan for the diagnosis of *Pneumocystis Jirovecii* Pneumonia in immunocompromised adults with acute respiratory failure

**DOI:** 10.1186/s13613-024-01337-8

**Published:** 2024-07-31

**Authors:** Laure Calvet, Virginie Lemiale, Djamel Mokart, Schellongowski Peter, Pickkers Peter, Alexande Demoule, Sangeeta Mehta, Achille Kouatchet, Jordi Rello, Philippe Bauer, Ignacio Martin-Loeches, Amelie Seguin, Victoria Metaxa, Magali Bisbal, Elie Azoulay, Michael Darmon

**Affiliations:** 1 Medical ICU, Saint-Louis University Hospital, AP-HP, 1 Avenue Claude Vellefaux, Paris, 75010 France; 2 Medical ICU, CHU Gabriel Montpied, Clermont-Ferrand, France; 3 Department of anesthesiology and Intensive Care, Institut Paoli-Calmettes, Marseille, France; 4 Department of Medicine I, Medical University of Vienna, Vienna, Austria; 5 The Department of Intensive Care Medicine (710), Radboud University Medical Center, Nijmegen, The Netherlands; 6 Medical ICU and Pneumology, Pitié-Salpétrière University Hospital, APHP, Paris, France; 7 Department of Medicine, Interdepartmental Division of Critical Care Medicine, Sinai Health System, University of Toronto, Toronto, Canada; 8 Intensive Care Unit, Angers University Hospital, Angers, France; 9 Centro de Investigacion Biomedica en Red en Enfermedades Respiratorias (CIBERES), Instituto de Salud Carlos III, Barcelona, Spain; 10 Clinical Research/Epidemiology In Pneumonia and Sepsis (CRIPS), Clinical Research, Vall d’Hebron Institute of Research (VHIR), CHU Nîmes, Barcelona, Nîmes, Spain; 11 Pulmonary and Critical Care Medicine, Mayo Clinic, Rochester, MN USA; 12 Department of Intensive Care Medicine, Multidisciplinary Intensive Care Research Organization (MICRO), St James Hospital, Dublin, Ireland; 13 Department of Clinical Medicine, Wellcome Trust‑HRB Clinical Research Facility, St. James’s Hospital, Trinity College, Dublin, Ireland; 14 Hospital de Barcelona, IDIBAPS, CIBERes, Barcelona, Spain; 15 Medical ICU, Nantes University Hospital, Nantes, France; 16 King’s College Hospital, SE5 9RS London, UK; 17 ECSTRA team, Biostatistics and clinical epidemiology, Université de Paris, UMR 1153 (center of epidemiology and biostatistic Sorbonne Paris Cité, CRESS), INSERM, Paris, France

**Keywords:** PCR, Beta-D glucan, *Pneumocystis* pneumonia; sensitivity, Specificity, Post test probability

## Abstract

**Background:**

The accuracy of a diagnostic test depends on its intrinsic characteristics and the disease incidence. This study aims to depict post-test probability of *Pneumocystis* pneumonia (PJP), according to results of PCR and Beta-D-Glucan (BDG) tests in patients with acute respiratory failure (ARF).

**Materials and methods:**

Diagnostic performance of PCR and BDG was extracted from literature. Incidence of *Pneumocystis* pneumonia was assessed in a dataset of 2243 non-HIV immunocompromised patients with ARF. Incidence of *Pneumocystis* pneumonia was simulated assuming a normal distribution in 5000 random incidence samples. Post-test probability was assessed using Bayes theorem.

**Results:**

Incidence of PJP in non-HIV ARF patients was 4.1% (95%CI 3.3-5). Supervised classification identified 4 subgroups of interest with incidence ranging from 2.0% (No ground glass opacities; 95%CI 1.4–2.8) to 20.2% (hematopoietic cell transplantation, ground glass opacities and no PJP prophylaxis; 95%CI 14.1–27.7). In the overall population, positive post-test probability was 32.9% (95%CI 31.1–34.8) and 22.8% (95%CI 21.5–24.3) for PCR and BDG, respectively. Negative post-test probability of being infected was 0.10% (95%CI 0.09–0.11) and 0.23% (95%CI 0.21–0.25) for PCR and BDG, respectively. In the highest risk subgroup, positive predictive value was 74.5% (95%CI 72.0-76.7) and 63.8% (95%CI 60.8–65.8) for PCR and BDG, respectively.

**Conclusion:**

Although both tests yield a high intrinsic performance, the low incidence of PJP in this cohort resulted in a low positive post-test probability. We propose a method to illustrate pre and post-test probability relationship that may improve clinician perception of diagnostic test performance according to disease incidence in predefined clinical settings.

**Supplementary Information:**

The online version contains supplementary material available at 10.1186/s13613-024-01337-8.

## Introduction

Biomarkers are used in various fields of medicine for screening and diagnosis, including prognostic or risk stratification [1–5]. Their usefulness and accuracy is dependent of intrinsic characteristics of the test (sensitivity, specificity) but also of the context in which the test is employed, as disease incidence influences extrinsic performance (positive and negative predictive value) [6]. Standards for Reporting of Diagnostic Accuracy Studies (STARD) guidelines, aiming to homogenize reporting of diagnostic performance assessment, underline the need to adequately assess studied population and to report extrinsic performance of tests [7]. Hence, impact of changes in pre-test probability over diagnostic test extrinsic performance is a well described mathematic correlation [8–10]. Nevertheless, pre-test probability may be difficult to assess [11], changes in post-test probability according to this later difficult to estimate, basic concepts of diagnostic test performance are poorly understood by medical students [10, 12], and usual indices of intrinsic performance may be misleadingly reassuring [13]. As a consequence, pre-test probability has been found to be taken into account infrequently by physicians when interpreting diagnostic test results [14, 15].

Alternative presentation of diagnostic test performance, including visual aids according to frequency has been advocated by some authors [10, 13]. *Pneumocystis* pneumonia in non-HIV patients might be suitable to reassess influence of pre-test probability on diagnostic test performance for several reasons: The disease is severe, and associated with a high morbidity and mortality [16–18]. The incidence of the disease is limited although context, clinical presentation, and radiological patterns may significantly changes pre-test probability [17, 19]. Last, available diagnostic tests, namely quantitative polymerase chain reaction (PCR) and β-D Glucan (BDG), have been described to have a good to very good intrinsic performance [20–23]. These tests are however quantitative, suggesting that intrinsic test performance may vary according to degree of positivity and adding further complexity in test interpretation [20–23]. These conditions not only underline needs for visual description for extrinsic test performance and potential clinical implications of findings, but also suggest that expert statements may be needed to help physician on daily basis.

The objective of this study was to assess incidence of *Pneumocystis* pneumonia in the general population of critically ill immunocompromised patients with acute respiratory failure, to detect subgroups of specific risk and to depict post-test probability of *Pneumocystis* pneumonia according to PCR and BDG tests in this setting.

## Methods

### Study population

In way to assess *Pneumocystis* pneumonia incidence, two distinct prospectively collected datasets were used.

The first set was the TRIALOH study dataset [24]. Patients were prospectively included from 2010 to 2012. The study was carried out in 17 university or university-affiliated centers in France and Belgium that belonged to a research network instituted in 2005. In all 17 centers, a senior intensivist and a senior hematologist were available around the clock and make ICU-admission decisions together. The appropriate ethics committees approved this study [24]. In this set, the attending physician assessed occurrence of acute respiratory failure prospectively and three independent experts reviewed all etiological diagnoses of acute respiratory failure. Since this study focuses on hematological patients with various reason for ICU admission, and to avoid artificially decreasing incidence of *Pneumocystis* pneumonia, only patients with Acute Respiratory Failure as the main reason for ICU admission were included in the current study.

The EFRAIM study was a multinational, observational prospective cohort study performed from Nov 2015 to July 2016 [25]. Investigators were critical care physicians from 16 countries with extensive experience in the management of various cohorts of critically ill immunocompromised patients. Participating providers obtained institutional review board (IRB) approval from their institutions in accordance with local ethics regulations. Only adult patients with acute respiratory failure, based upon predefined criterion were included in this study. All etiological diagnoses were reviewed by two study investigators for coherence and for alignment with established definitions [25].

### Definitions

#### Pneumocystis pneumonia

In both studies, etiologies of pulmonary involvement were diagnosed based on predefined criteria [26]. These criteria included type of immune defect, time between onset of the disease and ICU admission, radiologic presentation, and microbiological tests including direct search for *Pneumocystis* (direct examination with Gomorri-Grocott stain, immunostain, or PCR pneumocystis), and clinical course of patients. Beta-D-Glucan was uncommonly used in participating centers during both studies periods. For both included studies, study investigators reviewed *a posteriori* every diagnoses for coherence and alignment with established definitions (EFRAIM, TRIALOH).

*Ground glass opacities* were defined as any degree of ground glass opacities on CT-scan.

*Anti-Pneumocystis prophylaxis* was based on patients’ prescription before ICU admission without any regard to adherence.

*Lymphoid hematological malignancy* was defined as any acute or chronic underlying hematological malignancy including acute lymphoid leukemia, non-Hodgkin’s lymphoma, and chronic lymphoid leukemia.

*Hematopoietic Stem Cell Transplantation* (HSCT) was defined by any allogeneic or autologous stem cell transplantation independently of the conditioning protocol, origin or compatibility of donor cells, and without regard for delay since HSCT transplantation.

### Estimation of intrinsic performances of PCR and BD glucan by systematic literature review

A systematic review was performed on MEDLINE database using “*Pneumocystis* pneumonia (MeSH)” AND “sensitivity and specificity (MeSH)” AND/OR “(1–3)-β-D-Glucan” AND/OR “PCR” NOT “HIV (MeSH)”. Estimation of diagnostic test performance was validated by three authors (LC, VL, MD).

### Experts’ priors

Physicians’ priors were assessed before and after study results presentation. To do so, a standardized questionnaire assessing perception of priors with regard to incidence and post-test probability were obtained using visual analogic scale ranging from 0 to 100. Some data regarding experts’ characteristics were concomitantly obtained. Priors were searched for during a meeting of our research group (Groupe de Recherche en Réanimation Respiratoire et Onco-Hematologique). These meeting are held three time a year, contain both didactic presentations and presentation of study results focused on critically-ill cancer patients, with an attendance ranging from 50 to 100 intensivists. Responders were defined as being an expert if they presented on symposia or published in this field.

### Statistical analysis

Incidence and 95% confidence interval were computed by normal approximation in the general immunocompromised population with ARF.

To assess incidence and relevant clusters, a supervised tree partitioning was performed. Incidence and 95% confidence interval were computed in subgroup of interest identified by tree partitioning. Variables included in the supervised tree partitioning were age, gender, underlying immune defect, SOFA score, presence of ground glass opacities and preexisting prophylaxis.

Incidence and pre-test probability were then simulated in 5000 samples for the total cohort and subgroups identified by tree partitioning. These populations were simulated using a normal distribution centered on observed incidence and its confidence interval.

Diagnostic performance was modelized according to observed intrinsic performance, assuming binary results (positive vs. negative) and disregarding change in performance which may arise from quantitative analysis of either test or sample in which PCR is performed. Sensitivity and specificity were computed assuming a 0.5% uncertainty, following a continuous distribution and according to three hypotheses being, respectively:

Intermediate diagnostic performance: both sensitivity and specificity centered around the median observed performance for both PCR and BDG;

High sensitivity (weakly positive test): sensitivity was centered to the highest range of CI and specificity to the lowest range of the observed 95% confidence interval.

High specificity (highly positive test): sensitivity was centered to the lowest range of the observed CI and specificity to the lowest range of CI.

Post-test probability was computed using Bayes theorem and according for incidence and computed diagnostic test performance for the total cohort and the identified subgroups. Results are reported as median (IQR) and binary plot comparing pretest probability and post-test probability. In these plots, at a given pre-test probability, variability post-test probability reflect uncertainty regarding diagnostic test performance, and range of post-test probability uncertainty regarding true disease incidence. Post-test probability of successive concordant or discordant PCR and BDG was assessed assuming complete conditional independence of tests.

Last, in way to assess the influence of findings on physicians’ priors, densities of priors and difference between priors and findings were plotted before and after presentation of the results.

Analyses were performed using *R* software version 4.3.4 (R Project for Statistical Computing, Wien, Austria), *rpar*t and *infer* packages.

## Results

### Study population and pneumocystis pneumonia incidence

Of the 2622 critically ill immunocompromised patients included in the considered dataset, 2243 had an acute respiratory failure and were ultimately included in the current analysis. Median age was 62 years (51–70) and 1336 were of male gender (59.6%). The most common underlying malignancy were solid tumor in 774 patients (34.5%), acute myeloid leukemia in 382, non-Hodgkin’s Lymphoma in 358 patients (17.0%), Hodgkin’s lymphoma in 168 (7.5%), Myeloma in 121 (5.4%), and acute lymphoid leukemia in 89 (4.0%). 152 patients were allogeneic stem cell transplant recipients (6.8%) and 206 patients underwent autologous stem cell transplantation (9.2%). 378 patients were receiving anti-*Pneumocystis* prophylaxis at ICU admission (16.9%). Overall, 92 patients were considered having high probability *Pneumocystis* pneumonia (incidence 4.1%; 95% CI 3.8–4.4).

**Fig. 1:**
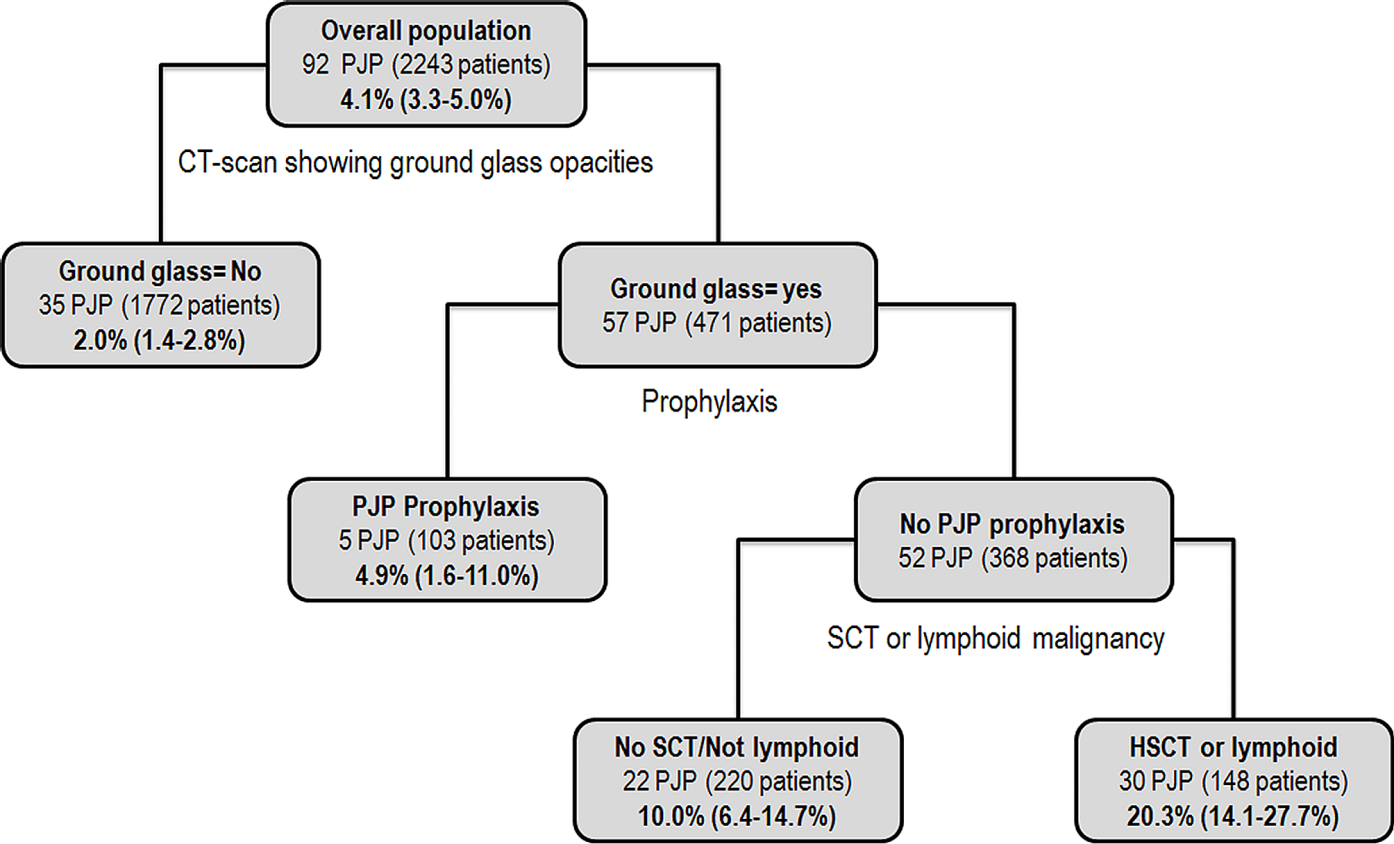
Tree reporting main clusters as regard to *Pneumocystis* pneumonia in the analyzed datasets (*n* = 2243)

Supervised tree partitioning identified four distinct subgroups of *Pneumocystis* pneumonia risk according to presence of (a) ground glass opacities at CT-scan; (b) anti-*Pneumocystis* prophylaxis at ICU admission, and (c) lymphoid underlying malignancy or previous hematopoietic stem cell transplantation (Fig. 1).

The observed *Pneumocystis* pneumonia incidences varied from 2.0% (95%CI 1.8–2.2) in patients without ground glass opacities to 20.3% (95% 18.3–22.2) in patients without prophylaxis, with underlying lymphoid leukemia or previous stem cell transplantation and with presence of ground glass opacities.

### Diagnostic test accuracy

After careful analysis of the literature, diagnostic test performance were set in accordance with systematic reviews reporting PCR and BDG test accuracy [21, 27]. For *Pneumocystis* PCR, we considered 98.3% sensitivity (95%CI 91.3–99.7) and 94.8% specificity (95% 90.8–97.1). For BDG, we considered 91.0% sensitivity (95%CI 82.7–95.5) and 86.3% specificity (95%CI 81.7–89.9). Cut-off of performance set for subsequent analyses are reported in table S1.

### Simulated incidence and post-test probability

The simulated incidence of of *Pneumocystis* pneumonia is reported in Tables 1 and 2.

**Fig. 2:**
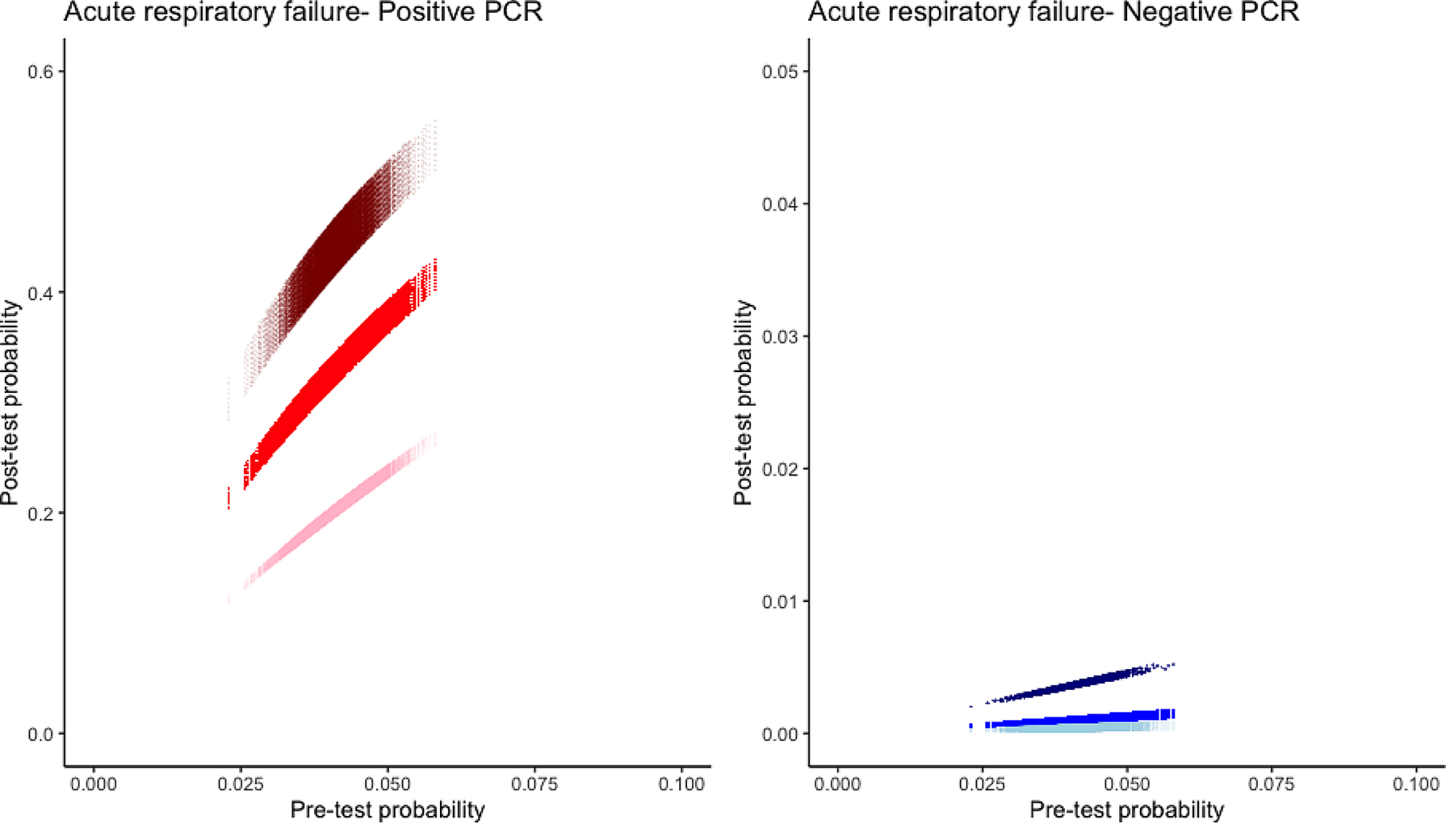
Relationship between incidence and post-test probability in the general population of immunocompromised patients with ARF and according to PCR result (positive = red, negative = blue). Diagnostic test performances are ranged from highly sensitive (light color) to sensitive test (dark color)

For intermediate diagnostic test performance, post-test probability if test was positive was 33% (95%CI 31.1–34.8) and 22.9% (95%CI 21.5–24.3) for PCR and BDG respectively (Figs. 1 and 2; Tables 1 and 2). Post-test probability if test was negative was 0.1% (95%CI 0.09–0.12) and 0.23% (95%CI 0.21–0.25) for PCR and BDG respectively (Figs. 1 and 2; Tables 1 and 2).

**Table 1:** Post-test probability of positive and negative PCR in non HIV critically ill patients with respiratory failure for the overall population and for the different subgroups of incidence

	General population	No ground glass	Ground glass and prophylaxis	Ground glass and no prophylaxis	Ground glass, no prophylaxis and lymphoid malignancy or HSCT
Incidence	4.1% [3.8–4.4]	2.0% [1.8–2.2]	4.8% [3.7–5.9]	10.0% [8.7–11.3]	20.3% [18.3–22.2]
			PCR +		
Specific test	44.0% [41.8–46.1]	27.4% [25.2–29.5]	48.2% [41.5–53.5]	67.1% [63.6–70.2]	82.3% [80.3–84.1]
Intermediate	33.0% [31.1–34.8]	19.0% [17.4–20.7]	36.8% [30.9–41.8]	56.1% [52.3–59.5]	74.4% [72.0-76.7]
Sensitive test	20.2% [18.8–21.4]	10.8% [9.8–11.8]	23.0% [18.6–26.9]	39.6% [36.0-42.9]	60.0% [56.9–62.8]
			PCR -		
Specific test	0.36% [0.33–0.39]	0.18% [0.16–0.20]	0.43% [0.33–0.54]	0.95% [0.81–1.09]	2.14% [1.89–2.41]
Intermediate	0.10% [0.09–0.12]	0.05% [0.04–0.06]	0.12% [0.09–0.15]	0.26% [0.22–0.31]	0.60% [0.51–0.70]
Sensitive test	0.04% [0.03–0.05]	0.02% [0.01–0.03]	0.05% [0.03–0.06]	0.10% [0.07–0.14]	0.24% [0.16–0.32]

**Table 2:** Post-test probability of positive and negative BDG in non HIV critically ill patients with respiratory failure for the overall population and for the different subgroups of incidence

	General population	No ground glass	Ground glass and prophylaxis	Ground glass and no prophylaxis	Ground glass, no prophylaxis and lymphoid malignancy or HSCT
Incidence	4.1% [3.8–4.4]	2.0% [1.8–2.2]	4.8% [3.7–5.9]	10.0% [8.7–11.3]	20.3% [18.3–22.2]
			BDG+		
Specific test	26.9% [25.3–28.9]	15.0% [13.7–16.3]	30.4% [25.0–35.0]	48.9% [45.1–52.3]	68.6% [65.8–71.1]
Intermediate	22.9% [21.5–24.3]	12.5% [11.4–13.6]	26.0% [21.2–30.3]	43.6% [39.9–47.0]	63.9% [60.8–66.5]
Sensitive test	18.9% [17.6–20.0]	10.0% [9.1–10.9]	21.5% [17.4–25.3]	37.6% [34.1–40.9]	58.0% [54.9–60.8]
			BDG -		
Specific test	0.41% [0.38–0.45]	0.20% [0.18–0.22]	0.49% [0.37–0.60]	1.06% [0.91–1.23]	2.40% [2.12–2.71]
Intermediate	0.23% [0.21–0.25]	0.11% [0.10–0.12]	0.27% [0.21–0.34]	0.60% [0.51–0.69]	1.36% [1.19–1.54]
Sensitive test	0.18% [0.16–0.19]	0.08% [0.07–0.09]	0.21% [0.16–0.26]	0.45% [0.39–0.53]	1.03% [0.90–1.18]

Performances according to risks clusters and test performance are depicted in Tables 1 and 2. In the highest risk group, post-test probability if test found positive ranged from 60.0 to 82.2% for PCR (Table 1). Similarly, post-test probability if test found positive ranged from 58.0 to 68.6% for BDG (Table 2).

Figures S1 and S2 report post-test probability according PCR results and subgroups. Figures S3 and S4 report post-test probability according PCR results and subgroups.

Figure S5 reports post-test probability after successive PCR and BDG testing assuming complete conditional independence of both tests.

### Intensivists’ priors

Overall, 25 physicians were interviewed before and after presentation of our results. Of them 5 had published a manuscript on *Pneumocystis* pneumonia or had been invited as speaker to our research group meeting and were considered experts. Median age of responders was 42 years (34–49). One expert reported conflict of interest related to *Pneumocystis* pneumonia.

Perception of *Pneumocystis* pneumonia and post-test probability of having the disease according to PCR and BDG were assessed in the general population of non-HIV immunocompromised patients with ARF (fig S6) and in the highest risk subgroup (fig S7). Before results presentation, perception systematically overestimated incidence in the general immunocompromised patients(+ 16% [IQR 6–16%]), in high risk subgroups and in post-test probability with both tests (+ 22% for PCR [IQR 7–37%] and + 27% for BDG [IQR 7–37%]; fig S5 and S6). This overestimation was found in both experts and non-experts (fig S8 and S9). Study results presentation resulted in decreased overestimation for incidence (+ 2% [IQR 1–6%]) and post-test probability with both tests (-3% for PCR [IQR − 10- -2%] and − 3% for BDG [IQR − 5–14%]. This decreased overestimation was observed in both experts and non-experts (Fig S5 to S8).

## Discussion

**Fig. 3:**
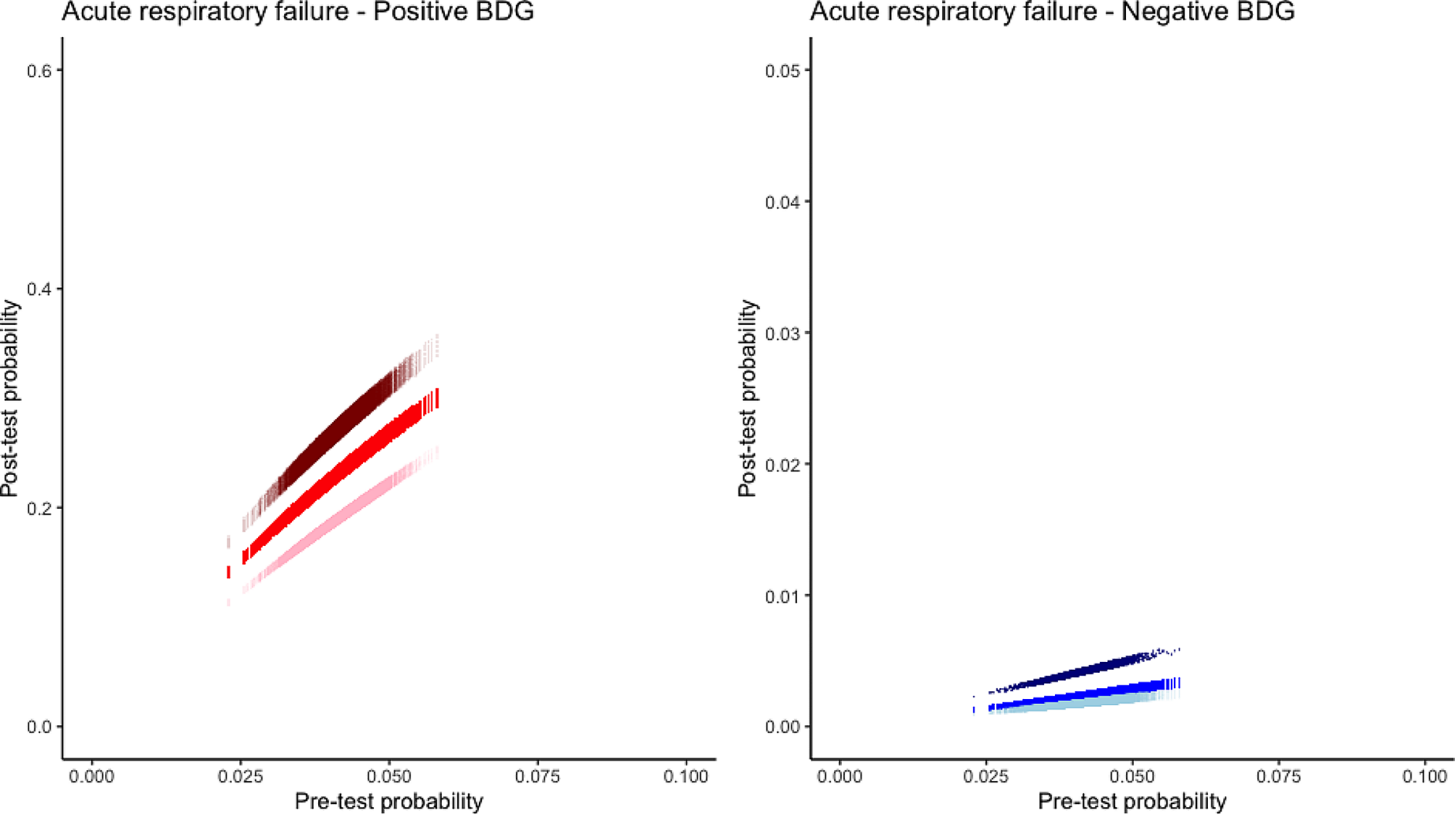
Relationship between incidence and post-test probability in the high-risk population of immunocompromised patients with ARF and according to PCR result (positive = red, negative = blue). Diagnostic test performance are ranged from highly sensitive (light color) to sensitive test (dark color)

Our study is the first to the best of our knowledge to assess incidence of *Pneumocystis* in way to delineate post-test probability of disease in identified subgroups. According to our results, despite assumption of a high sensitivity and specificity, positive post-test probability of *Pneumocystis* pneumonia is limited after positive PCR or BDG test as consequences of the limited incidence of the disease in immunocompromised patients. We propose a visual representation (Figs. 2 and 3) that may help physician to appreciate interaction between observed incidence and intrinsic diagnostic test performance at bedside while taking into account incidence and diagnostic test performance uncertainties.

In line with previous studies, the incidence of *Pneumocystis* pneumonia is low in the population of critically ill immunocompromised patients admitted for an acute respiratory failure [17, 25, 28]. In line with previous studies, identified risk factors of *Pneumocystis* pneumonia were preexisting lymphoid disease or stem cell transplantation, ground glass opacities at CT scan and lack of *Pneumocystis* prophylaxis [17, 29]. In this large cohort of patients suggest incidence ranging from 2% (1.4-2.8%) in lowest risk subgroup to 20.2% (17.8-27.8%) in the high risk subgroup.

Not surprisingly, even assuming excellent intrinsic performance of *Pneumocystis* PCR and BDG, our results underline that the poor positive post-test probability resulting from the low incidence translates into high risk of false positive diagnosis and, more importantly, failure to identify culprit of the ARF with potentially negative consequences [19, 25, 30].

Interestingly, although arising from simulation data, our attempt to depict graphically relationship between incidence in risk subgroup and post-test probability translated into dramatic change in perception of disease incidence, diagnostic performance of the test in this setting and ultimately perception of potential significance of diagnostic test [12, 31]. Previous studies underlined frequent physicians’ misinterpretation of disease incidence [32, 33], frequent misperception and overestimation of diagnostic test performance [10, 34, 35] and most importantly a limited comprehension of pre-test/post-test relationship and its implication at bedside [10, 34, 35]. Thus, when facing various hypotheses of disease incidence, previous studies suggested a lack of perception of changes in post-test probability by physicians [14]. In addition, previous studies suggested misperception to persist even when diagnostic test performance were described as likelihood ratios rather than as sensitivity and specificities, these later being independent of disease incidence ^13^. Thus, previous studies suggested use of natural frequency of disease to allow better perception of diagnostic test performance [10], short course of Bayesian reasoning to partly improved this perception [12] and, best of all, graphical representation to allow improved understanding of diagnostic test performance (32). Although these results may deserve to be confirmed, our depiction of the pre-test / post-test relationship in various subgroups may help in depicting accurately a known and mathematical relationship, may help physician in apprehending input of positive or negative test in various subgroup or clinical scenarios, while accounting for uncertainty both in term of diagnostic test performance and in disease incidence.

This study has several limitations. First, we aimed in assessing incidence and depicting incidence/post-test probability in various predefined subgroups. Although this approach may make sense at a population level, it disregards fact that for a given patient, pre-test probability ranges from 0 to 1 and cannot be limited to the incidence of the disease. Hence, several subgroup of interest were not tested including stratification according to duration of symptoms before ICU admission, type of anti-*Pneumocystis* prophylaxis, or specific symptoms of ARF. In this line, solid organ recipients were under-represented which may have influenced our results and results of the high-risk group may have been modified by emerging targeted therapies. Our results may however help in identifying subgroup of patients in whom tests may be useless or at least should be interpreted only if negative. This limit suggests additional studies in specific subgroups may be required to refine our results and improve overall view of pre-test probability. Last, although we tried to depict impact of successive test, these results are probably misleading. Hence, both test are likely to be highly correlated, and covariance of both test has never been assessed to the best of our knowledge. As consequences, performance of concordant PCR and BDG in our study probably overestimate post-test probability and dedicated studies are needed.

Moreover, diagnoses of *Pneumocystis* pneumonia in the initial dataset were confirmed in most cases using either PCR and/or BDG test, in specific setting and after expert validation. Although this could have impaired assessment of diagnostic test performance, we only used these data to set range of incidence and not to validate intrinsic test performance of the test, this limit being unlikely to have influenced our findings. Furthermore, we lack validated gold standard in confirming *Pneumocystis* pneumonia. Therefore, incidence in the study population may have been overestimated. This bias however strengthens our findings, lower incidence translating into lower positive post-test probability. Last, although we validated our results when compared to experts’ priors, no validation against existing standards was performed. Thus, whether our visual representation may perform better than classical Fagan’s nomogram with usual pre-test probability range underlined (example given as figure S9) may deserve to be assessed in future studies.

## Conclusion

In this study we hypothesized and validated that despite excellent intrinsic performance, both *Pneumocystis* PCR and BDG displayed a limited positive predictive value in critically ill immunocompromised patients with acute respiratory failure. This analysis underlines need for adequate pre-test estimation of probability of *Pneumocystis* pneumonia to allow interpretation of laboratory tests results. We display a visual representation that may help physician to understand influence of observed incidence on post-test probability of a disease and be a first step implement clinical vignette to underline case-scenario in which these tests might be relevant.

### Electronic supplementary material

Below is the link to the electronic supplementary material.

Supplementary Material 1

## Data Availability

The datasets used during the current study are available from the corresponding author on reasonable request.

## Abbreviations

ARF: Acute respiratory failure
BDG: Beta-D glucan
CI: Confidence interval
HSCT: Hematopoietic stem cell transplantation
ICU: Intensive care unit
IQR: Interquartile range
IRB: Institutional review board
OR: Odds ratio
PCR: Polymerase chain reaction
PPV: Positive predictive value

## References

[1] Bouadma L, Luyt C-E, Tubach F, et al. Use of procalcitonin to reduce patients’ exposure to antibiotics in intensive care units (PRORATA trial): a multicentre randomised controlled trial. Lancet Lond Engl. 2010;375(9713):463–74. 10.1016/S0140-6736(09)61879-1 20097417

[2] Bihorac A, Chawla LS, Shaw AD, et al. Validation of cell-cycle arrest biomarkers for acute kidney injury using clinical adjudication. Am J Respir Crit Care Med. 2014;189(8):932–9. 24559465 10.1164/rccm.201401-0077OC

[3] McDonald AC, Vira M, Shen J, et al. Circulating microRNAs in plasma as potential biomarkers for the early detection of prostate cancer. Prostate. 2018;78(6):411–8. 29383739 10.1002/pros.23485

[4] Shlipak MG, Matsushita K, Ärnlöv J, et al. Cystatin C versus creatinine in determining risk based on kidney function. N Engl J Med. 2013;369(10):932–43. 24004120 10.1056/NEJMoa1214234 PMC3993094

[5] Riedlinger D, Möckel M, Müller C, et al. High-sensitivity cardiac troponin T for diagnosis of NSTEMI in the elderly emergency department patient: a clinical cohort study. Biomark Biochem Indic Expo Response Susceptibility Chem. 2018;23(6):551–7. 10.1080/1354750X.2018.1460763 29619842

[6] Florkowski CM. Sensitivity, specificity, receiver-operating characteristic (ROC) curves and likelihood ratios: communicating the performance of diagnostic tests. Clin Biochem Rev. 2008;29(Suppl 1):S83–87. 18852864 PMC2556590

[7] Bossuyt PM, Reitsma JB, Bruns DE, et al. STARD 2015: an updated list of essential items for reporting diagnostic accuracy studies. BMJ. 2015;351:h5527. 26511519 10.1136/bmj.h5527 PMC4623764

[8] Kallner A. Bayes’ theorem, the ROC diagram and reference values: definition and use in clinical diagnosis. Biochem Med. 2018;28(1):010101. 10.11613/BM.2018.010101 PMC5707117 29209139

[9] Diamond GA, Forrester JS, Hirsch M, et al. Application of conditional probability analysis to the clinical diagnosis of coronary artery disease. J Clin Invest. 1980;65(5):1210–21. 6767741 10.1172/JCI109776 PMC371455

[10] Whiting PF, Davenport C, Jameson C, et al. How well do health professionals interpret diagnostic information? A systematic review. BMJ Open. 2015;5(7):e008155. 26220870 10.1136/bmjopen-2015-008155 PMC4521525

[11] Richardson WS. Five uneasy pieces about pre-test probability. J Gen Intern Med. 2002;17(11):882–3. 12406361 10.1046/j.1525-1497.2002.20916.x PMC1495129

[12] Noguchi Y, Matsui K, Imura H, Kiyota M, Fukui T. Quantitative evaluation of the diagnostic thinking process in medical students. J Gen Intern Med. 2002;17(11):839–44. 12406355 10.1046/j.1525-1497.2002.20139.x PMC1495132

[13] Moreira J, Bisoffi Z, Narváez A, Van den Ende J. Bayesian clinical reasoning: does intuitive estimation of likelihood ratios on an ordinal scale outperform estimation of sensitivities and specificities? J Eval Clin Pract. 2008;14(5):934–40. 19018928 10.1111/j.1365-2753.2008.01003.x

[14] Agoritsas T, Courvoisier DS, Combescure C, Deom M, Perneger TV. Does prevalence matter to physicians in estimating post-test probability of disease? A randomized trial. J Gen Intern Med. 2011;26(4):373–8. 21053091 10.1007/s11606-010-1540-5 PMC3055966

[15] Puhan MA, Steurer J, Bachmann LM, Riet G. ter. A randomized trial of ways to describe test accuracy: the effect on physicians’ post-test probability estimates. *Ann Intern Med* 2005;143(3):184–189. 10.7326/0003-4819-143-3-200508020-00004 16061916

[16] Bollée G, Sarfati C, Thiéry G, et al. Clinical picture of Pneumocystis Jiroveci pneumonia in cancer patients. Chest. 2007;132(4):1305–10. 17934116 10.1378/chest.07-0223

[17] Roux A, Canet E, Valade S, et al. Pneumocystis Jirovecii pneumonia in patients with or without AIDS, France. Emerg Infect Dis. 2014;20(9):1490–7. 25148074 10.3201/eid2009.131668 PMC4178412

[18] Zahar JR, Robin M, Azoulay E, Fieux F, Nitenberg G, Schlemmer B. Pneumocystis carinii pneumonia in critically ill patients with malignancy: a descriptive study. Clin Infect Dis off Publ Infect Dis Soc Am. 2002;35(8):929–34. 10.1086/342338 12355379

[19] Azoulay E, Lemiale V, Mokart D, et al. Acute respiratory distress syndrome in patients with malignancies. Intensive Care Med. 2014;40(8):1106–14. 24898895 10.1007/s00134-014-3354-0

[20] Alanio A, Desoubeaux G, Sarfati C, et al. Real-time PCR assay-based strategy for differentiation between active Pneumocystis jirovecii pneumonia and colonization in immunocompromised patients. Clin Microbiol Infect off Publ Eur Soc Clin Microbiol Infect Dis. 2011;17(10):1531–7. 10.1111/j.1469-0691.2010.03400.x 20946413

[21] Fan L-C, Lu H-W, Cheng K-B, Li H-P, Xu J-F. Evaluation of PCR in bronchoalveolar lavage fluid for diagnosis of Pneumocystis Jirovecii pneumonia: a bivariate meta-analysis and systematic review. PLoS ONE. 2013;8(9):e73099. 24023814 10.1371/journal.pone.0073099 PMC3762835

[22] Onishi A, Sugiyama D, Kogata Y, et al. Diagnostic accuracy of serum 1,3-β-D-glucan for pneumocystis jiroveci pneumonia, invasive candidiasis, and invasive aspergillosis: systematic review and meta-analysis. J Clin Microbiol. 2012;50(1):7–15. 22075593 10.1128/JCM.05267-11 PMC3256688

[23] Sun W, Wang K, Gao W, et al. Evaluation of PCR on bronchoalveolar lavage fluid for diagnosis of invasive aspergillosis: a bivariate metaanalysis and systematic review. PLoS ONE. 2011;6(12):e28467. 22164295 10.1371/journal.pone.0028467 PMC3229594

[24] Azoulay E, Mokart D, Pène F, et al. Outcomes of critically ill patients with hematologic malignancies: prospective multicenter data from France and Belgium–a groupe de recherche respiratoire en réanimation onco-hématologique study. J Clin Oncol off J Am Soc Clin Oncol. 2013;31(22):2810–8. 10.1200/JCO.2012.47.2365 23752112

[25] Azoulay E, Pickkers P, Soares M, et al. Acute hypoxemic respiratory failure in immunocompromised patients: the Efraim multinational prospective cohort study. Intensive Care Med. 2017;43(12):1808–19. 28948369 10.1007/s00134-017-4947-1

[26] Azoulay E, Mokart D, Lambert J, et al. Diagnostic strategy for hematology and oncology patients with acute respiratory failure: randomized controlled trial. Am J Respir Crit Care Med. 2010;182(8):1038–46. 20581167 10.1164/rccm.201001-0018OC

[27] Karageorgopoulos DE, Qu J-M, Korbila IP, Zhu Y-G, Vasileiou VA, Falagas ME. Accuracy of β-D-glucan for the diagnosis of Pneumocystis Jirovecii pneumonia: a meta-analysis. Clin Microbiol Infect off Publ Eur Soc Clin Microbiol Infect Dis. 2013;19(1):39–49. 10.1111/j.1469-0691.2011.03760.x 22329494

[28] Neofytos D, Hirzel C, Boely E, et al. Pneumocystis Jirovecii pneumonia in solid organ transplant recipients: a descriptive analysis for the Swiss transplant cohort. Transpl Infect Dis off J Transpl Soc. 2018;20(6):e12984. 10.1111/tid.12984 30155950

[29] Azoulay E, Roux A, Vincent F et al. A multivariable prediction model for Pneumocystis Jirovecii Pneumonia in Hematology patients with Acute Respiratory failure. Am J Respir Crit Care Med 2018. 10.1164/rccm.201712-2452OC 29995433

[30] Contejean A, Lemiale V, Resche-Rigon M, et al. Increased mortality in hematological malignancy patients with acute respiratory failure from undetermined etiology: a Groupe De Recherche en Réanimation respiratoire en Onco-Hématologique (Grrr-OH) study. Ann Intensive Care. 2016;6(1):102. 27783381 10.1186/s13613-016-0202-0 PMC5080277

[31] Visual representation of statistical information improves diagnostic inferences in doctors and their patients. - PubMed - NCBI [Internet]. [cited 2019 Sep 11]; https://www.ncbi.nlm.nih.gov/pubmed/?term=Garcia-Retamero+Soc+Sci+Med.+2013.

[32] Poses RM, Cebul RD, Collins M, Fager SS. The accuracy of experienced physicians’ probability estimates for patients with sore throats. Implications for decision making. JAMA. 1985;254(7):925–9. 3894705 10.1001/jama.1985.03360070063024

[33] Poses RM, Anthony M. Availability, wishful thinking, and physicians’ diagnostic judgments for patients with suspected bacteremia. Med Decis Mak Int J Soc Med Decis Mak. 1991;11(3):159–68. 10.1177/0272989X9101100303 1881270

[34] Lyman GH, Balducci L. Overestimation of test effects in clinical judgment. J Cancer Educ off J Am Assoc Cancer Educ. 1993;8(4):297–307. 10.1080/08858199309528246 8186081

[35] Eddy DM. Probabilistic reasoning in clinical medicine: Problems and opportunities [Internet]. Judgm. Uncertain. Heuristics Biases. 1982 [cited 2019 Sep 11.]

