# Supplementary material for: Interpretation of results of PCR and B-D-glucan for the diagnosis of Pneumocystis Jirovecii Pneumonia in immunocompromised adults with acute respiratory failure
Source: Ann Intensive Care. 2024 Jul 31;14:120. doi: 10.1186/s13613-024-01337-8 (PMC11291821; doi:10.1186/s13613-024-01337-8)
Supplement: Supplementary file 1 — Supplementary Material 1 [file 13613_2024_1337_MOESM1_ESM.docx]

**Diagnostic Accuracy of PCR and B-D-glucan for the Diagnosis of Pneumocystis Jirovecii Pneumonia in immunocompromised patients with acute respiratory failure**

Laure CALVET et al.

Supplementary appendix

**Study investigators:**

**Efraim investigators and the Nine-I study group – Contributors to the study**

| **First Name** | **Last Name** | **Affiliation** | **City** | **Country** |
| --- | --- | --- | --- | --- |
| Antoine | Rabbat | Hôpital Cochin | Paris | France |
| Isabelle | Vinatier | CHD de Vendée | La Roche Sur Yon | France |
| Michael | Darmon | CHU | Saint-Etienne | France |
| Kada | Klouche | CHU | Montpellier | France |
| Laura | Platon | CHU | Montpellier | France |
| Martine | Nyunga | CHG Victor Provo | Roubaix | France |
| Julien | Mayaux | CHU Pitié-Salpétrière | Paris | France |
| Florent | Wallet | CHU | Lyon Sud | France |
| Akli | Chermak | CH Sud Essonne | Etampes | France |
| Amelie | Seguin | Réanimation Medicale - CHU de Caen | Caen | France |
| Caroline | Lemaitre | University Hospital, Medical Intensive Care | Rouen | France |
| Elise | Artaud-Macari | University Hospital, Medical Intensive Care | Rouen | France |
| Jonas | Nelsen | Rigshospitalet, | Copenhagen | Denmark |
| Ann M. | Moeller | Herlev university hospital, UCPH | Herlev | Denmark |
| Thomas | Kaufmann | Department of Critical Care | Groningen | Netherlands |
| Dennis | Bergmans | Department of Critical Care | Maastricht | Netherlands |
| Angélique | Spoelstra – de Man | Department of Critical Care | Amsterdam | Netherlands |
| Ana Paula | Pierre de Moraes | Hospital de Câncer do Maranhao |  | Brazil |
| William | Viana | Hospital Copa d'Or | Rio de Janeiro | Brazil |
| Guilliana | Moralez | Hospital GetulioVargas | Rio de Janeiro | Brazil |
| Thiago | Lishoa | Hospital Santa Rita, Santa Casa de Misericordia | Porte Allegre | Brazil |
| Thiago Domingos | Correa | ICU, Hospital Israelita Albert Einstein | São Paulo | Brazil |
| Belen | Encina | Critical Care, Hospital Quiron | Barcelona | Spain |
| Gabriel | Moreno | Department of Critical Care, Bellvitge Hospital | Barcelona | Spain |
| Emilio | Rodriguez | Department of Critical Care | Santiago de Compostela | Spain |
| Llorenç | Socias-Crespi | Department of Critical Care, H. Son Llacer | Palma | Spain |
| Yadav | Hemang | Pulmonary and Critical Care Medicine, Mayo Clinic | Rochester | USA |
| Anne-Pascale | Meert | Institut Jules Bordet | Brussels | Belgique |
| Dominique | Benoit | Ghent University Hospital | Ghent | Belgique |
| Nina | Buchtele | Department of Medicine I | Vienna | Austria |
| Thomas | Staudinger | Department of Medicine I | Vienna | Austria |
| Gottfried | Heinz | Department of Medicine II | Vienna | Austria |
| Gürkan | Sengölge | Department of Medicine III | Vienna | Austria |
| Christian | Zauner | Department of Medicine III | Vienna | Austria |
| Peter | Jaksch | Department of Thoracic Surgery | Vienna | Austria |
| Karin | Amrein | Department of Internal Medicine | Graz | Austria |
| Aisling | Mc Mahon | Department of Critical Care, St James, | Dublin | Ireland |
| Brian | Marsh | Department of Critical Care, Mater misericordia | Dublin | Ireland |
| Martin | Balik | Department of Critical Care | Prague | Czech republic |
| Thomas | Karvunidis | Department of Critical Care | Pielsa | Czech republic |
| Pål | Klepstad | St. Olavs Hospital | Trondheim | Norway |
| Anne | Kuitunen | Department of Critical Care | Tempere | Finland |
| Gilda | Cinnella | Ospedali Riuniti, Department of Critical Care | Foggia | Italy |
| Antonella | Cotoia | Ospedali Riuniti, Department of Critical Care | Foggia | Italy |
| Sumech | Shah | Mount Sinai Hospital | Toronto | Canada |

**Acknowledgments**

This study was performed on behalf of the “Caring for critically ill immunocompromised patients – Multinational Network (Nine-I). This group includes critical care specialists from 16 countries in Europe, USA, Canada and South America. The primary aim of this group is to improve and standardize practices in the management of critically ill immunocompromised patients.

**TRIALOH Investigators**

**Jérôme Lambert, Louise-Marie Laisne, Marine Chaize, Benoit Schlemmer, Sylvie Chevret:** Saint-Louis University Hospital, France

**Frédéric Pène :** Cochin University Hospital, France

**Julien Mayaux :** Pitié-Salpétrière University Hospital, France

**Antoine Rabbat :** Hôtel Dieu University Hospital, France

**Achille Kouatchet :** Centre Hospitalier Universitaire, Angers, France

**François Vincent :** Avicenne University Hospital, Bobigny, France

**Martine Nyunga :** Victor Provo Hospital, Roubaix, France

**Fabrice Bruneel :** Mignot Hospital, Versailles, France

**Christine Lebert :** Montaigu Hospital, La Roche sur Yon, France

**Pierre Perez :** Brabois University Hospital, Nancy, France

**Anne Renault :** Brest University Hospital, Brest, France

**Rebecca Hamidfar :** Albert Michallon University Hospital, Grenoble, France

**Mercé Jourdain :** Salengro University Hospital, Lille, France

**Anne-Pascale Meert:** Institut Jules Bordet, Brussels, Belgium

**Dominique Benoit:** Ghent University Hospital, Ghent, Belgium

**Table S1.** Intrinsic performance assumptions for PCR and BDG

|  | **Sensitivity** | **Specificity** |
| --- | --- | --- |
| PCR Pneumocystis | | |
| Specific test | 91.8% [91.5-92.1] | 95.0% [94.8-95.3] |
| Intermediate | 97.8% [97.6-98.05] | 91.5% [91.2-91.8] |
| Sensitive test | 99.2% [99.0-99.5] | 83.2% [83.0-83.6] |
| B-D-Glucan | | |
| Specific test | 91.3% [91.1-91.6] | 89.4% [89.2-89.7] |
| Intermediate | 95.3% [95.1-95.6] | 86.3% [86.1-86.5] |
| Sensitive test | 96.7% [96.4-96.9] | 82.2% [82.0-82.5] |

**Figure S1.** Relationship between incidence and post-test probability after positive pneumocystis PCR in the overall population and in different risk subgroups


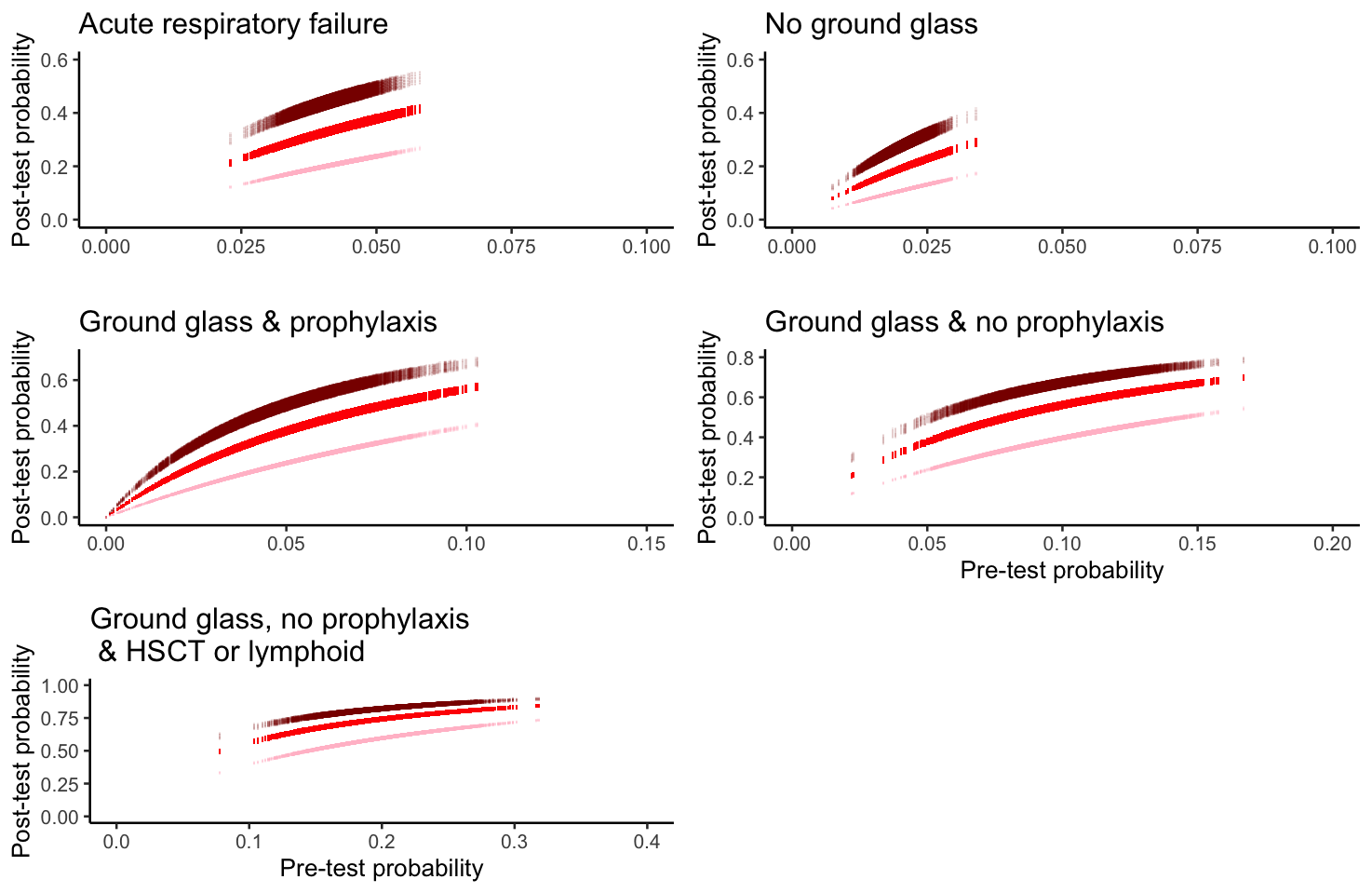


**Figure S2.** Relationship between incidence and post-test probability after negative pneumocystis PCR in the overall population and in different risk subgroups


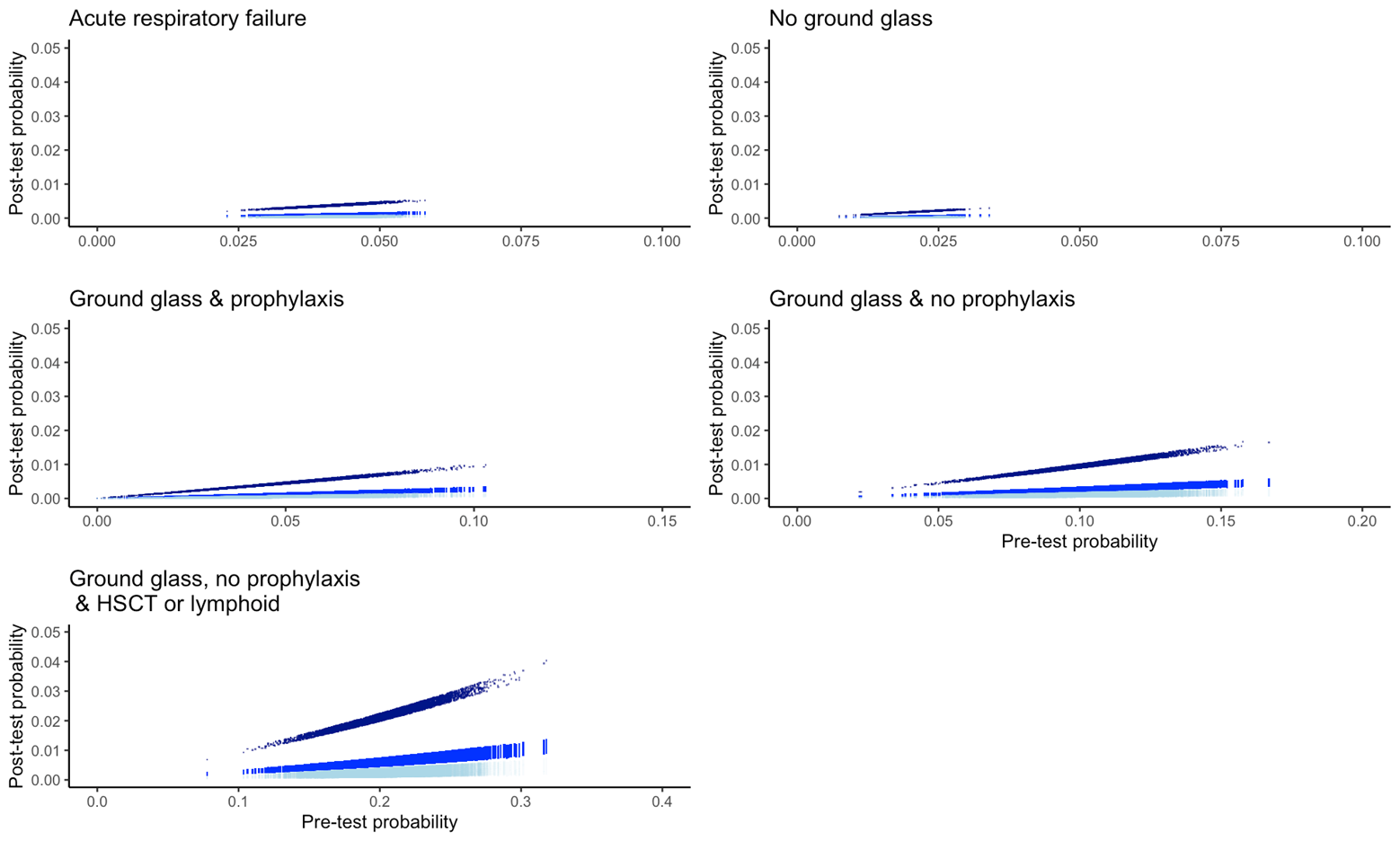


**Figure S3.** Relationship between incidence and post-test probability after positive pneumocystis BDG test in the overall population and in different risk subgroups


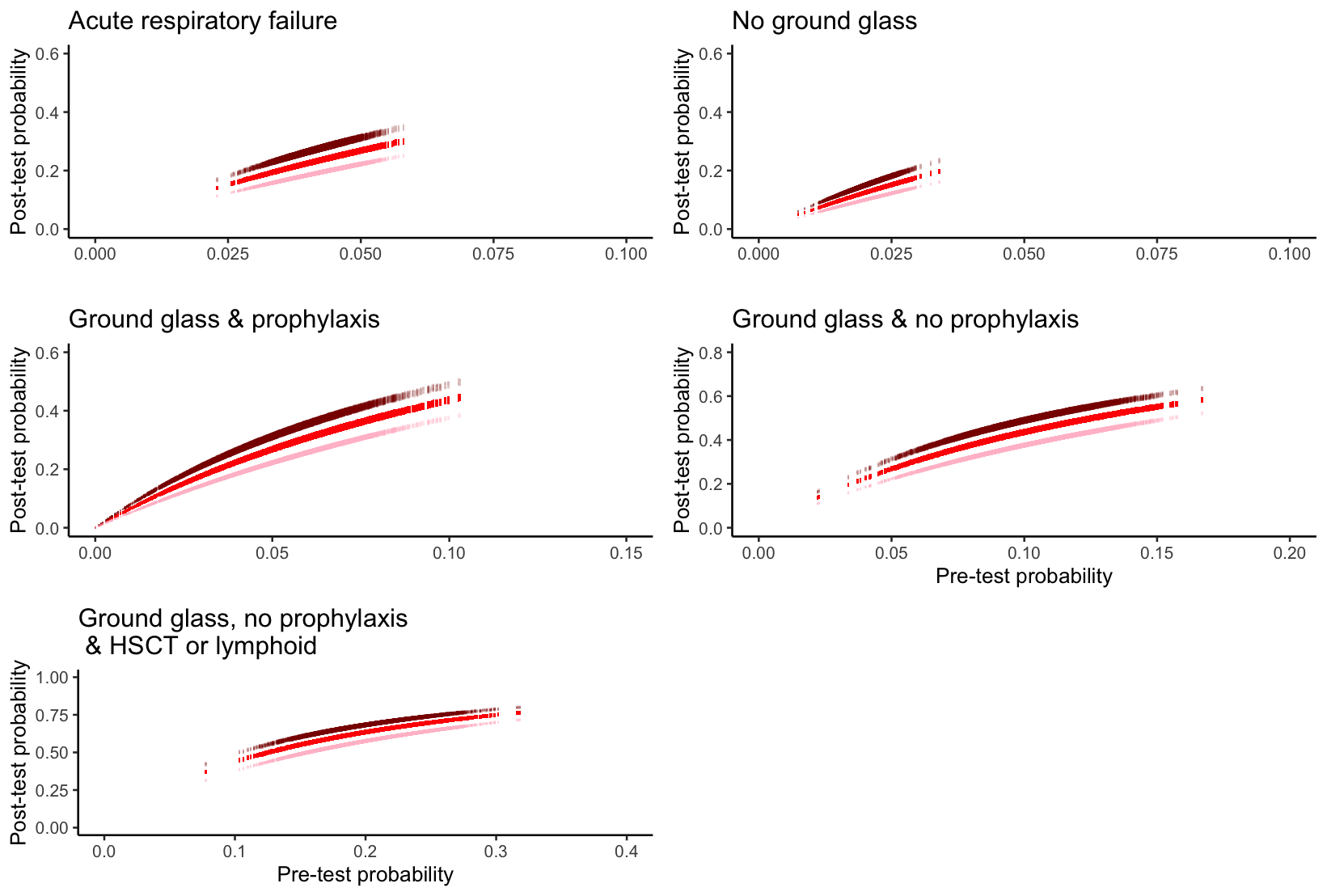


**Figure S4.** Relationship between incidence and post-test probability after negative BDG test in the overall population and in different risk subgroups


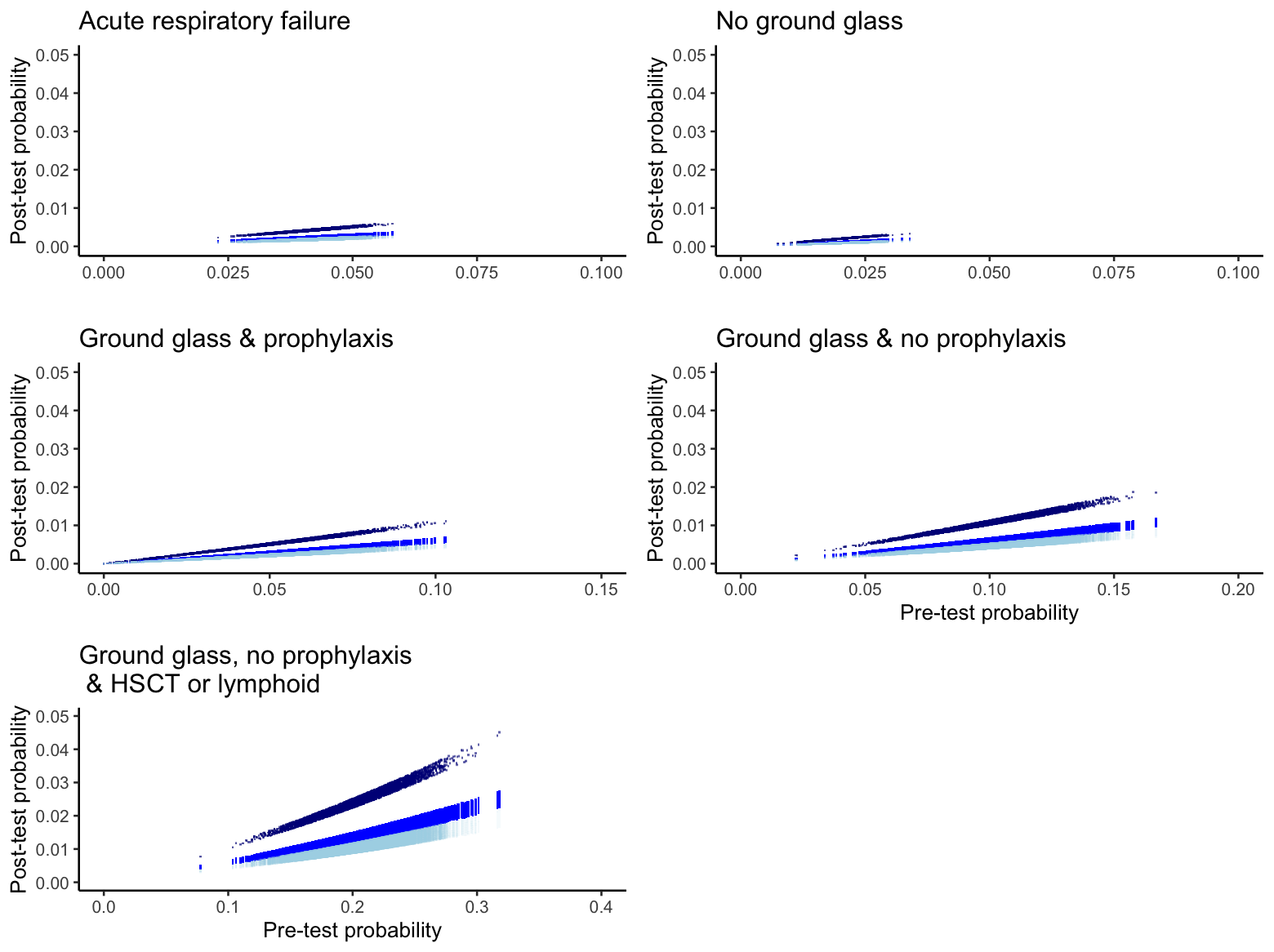


**Figure S5.** Relationship between incidence and post-test probability after successive tests (PCR and BDG) assuming conditional independence of the test in the overall population of non-HIV immunocompromised patients with acute respiratory failure.


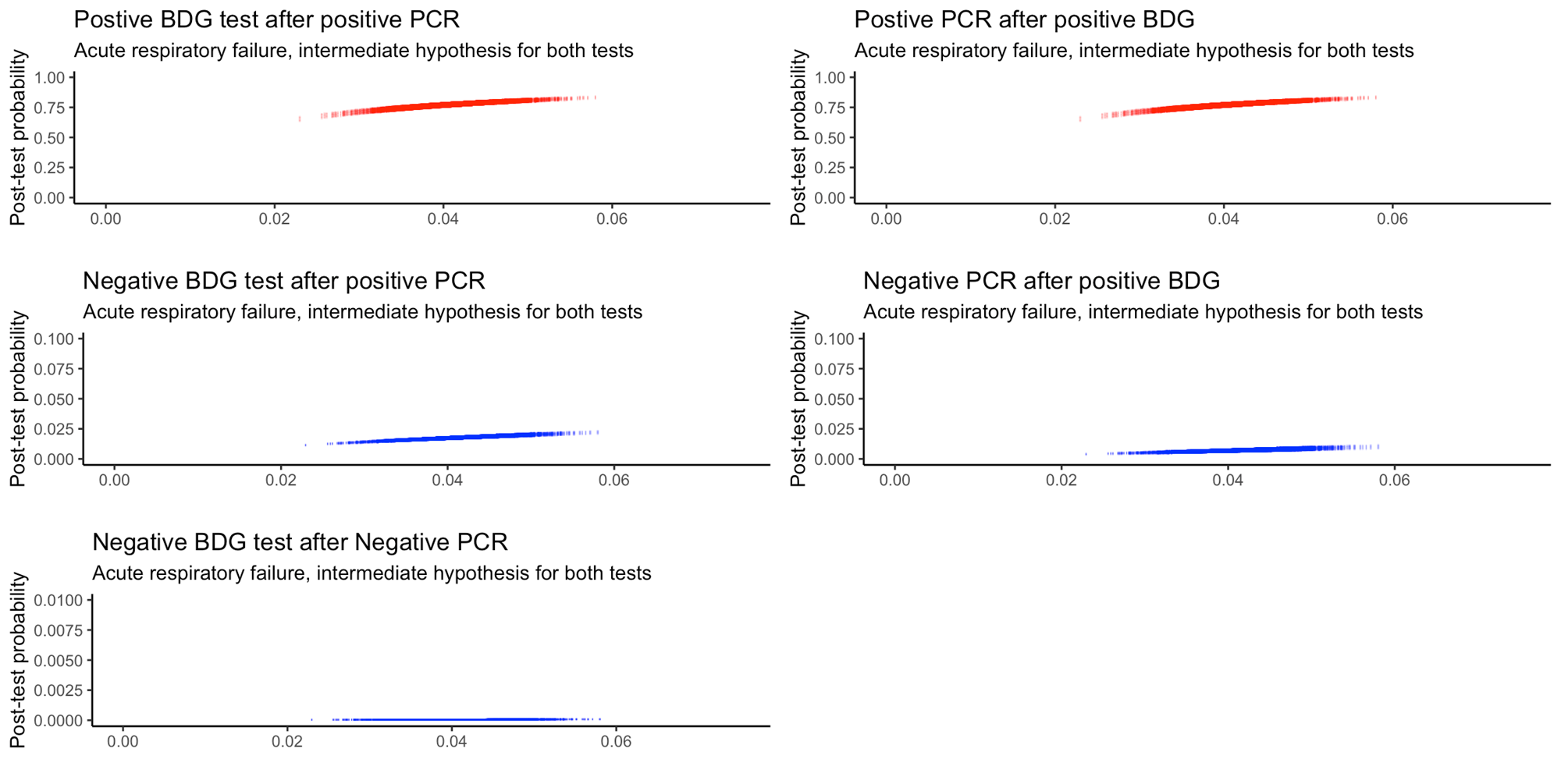


**Figure S6.** Pre/post evaluation of physicians’ priors as regard to incidence of pneumocystis pneumonia and predictive value of PCR and BD glucan test in the overall population of critically ill immunocompromised patients with acute respiratory failure


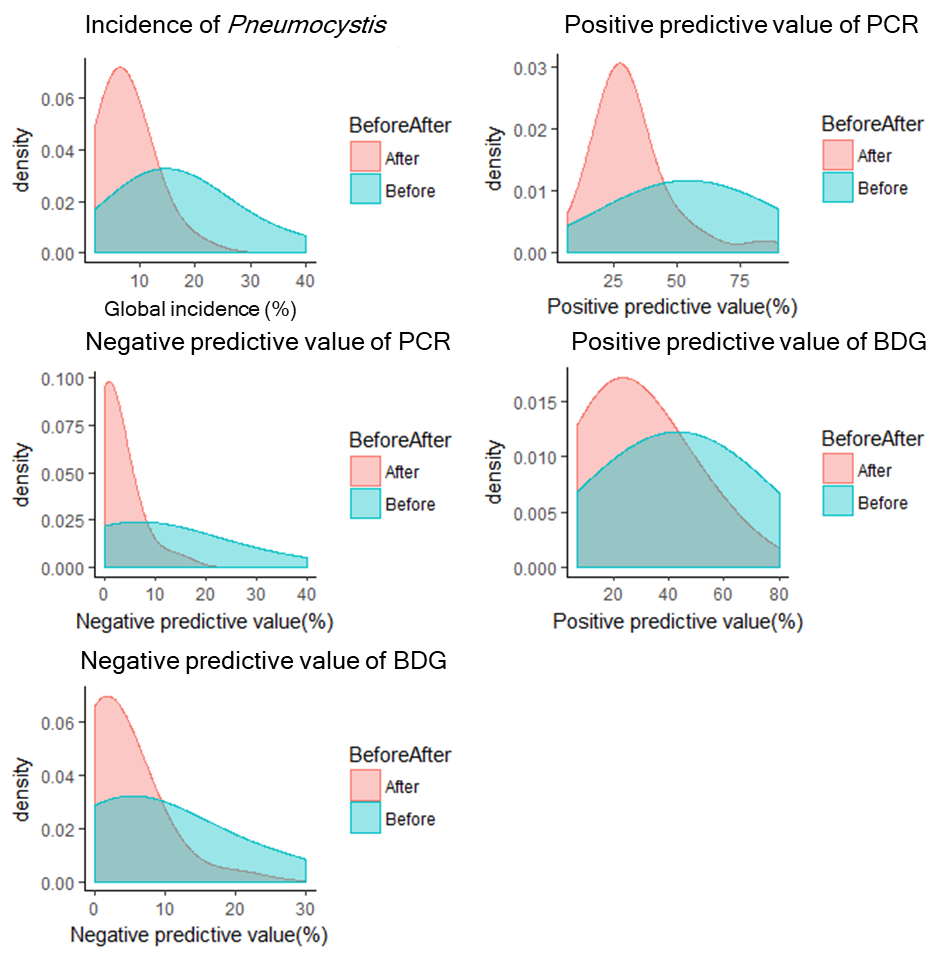


**Figure S7.** Pre/post evaluation of physicians’ priors as regard to incidence of pneumocystis pneumonia and predictive value of PCR and BD glucan test in the high risk subgroup of critically ill immunocompromised patients with acute respiratory failure (i.e. with ground glass opacities, with lymphoid malignancy or HSCT and w/o prophylaxis).


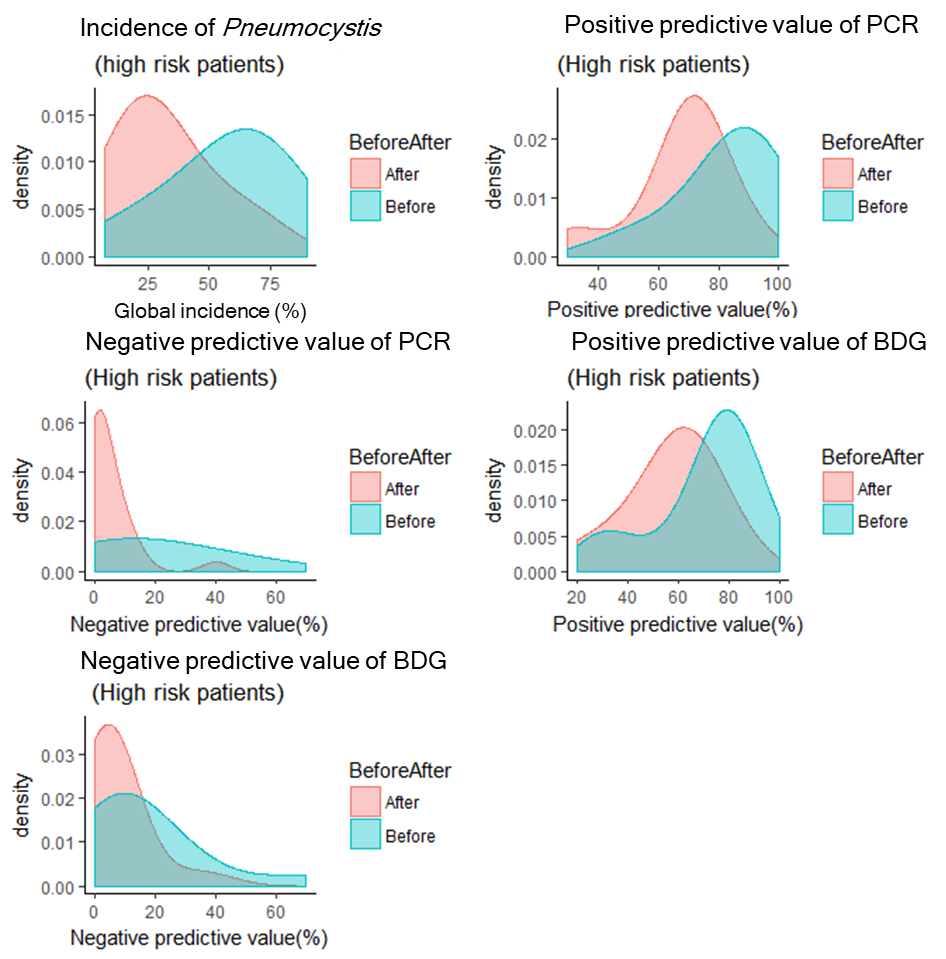


**Figure S8.** Pre/post evaluation of expert and non-expert physicians’ priors as regard to incidence of pneumocystis pneumonia and predictive value of PCR and BD glucan test in the overall population of critically ill immunocompromised patients with acute respiratory failure


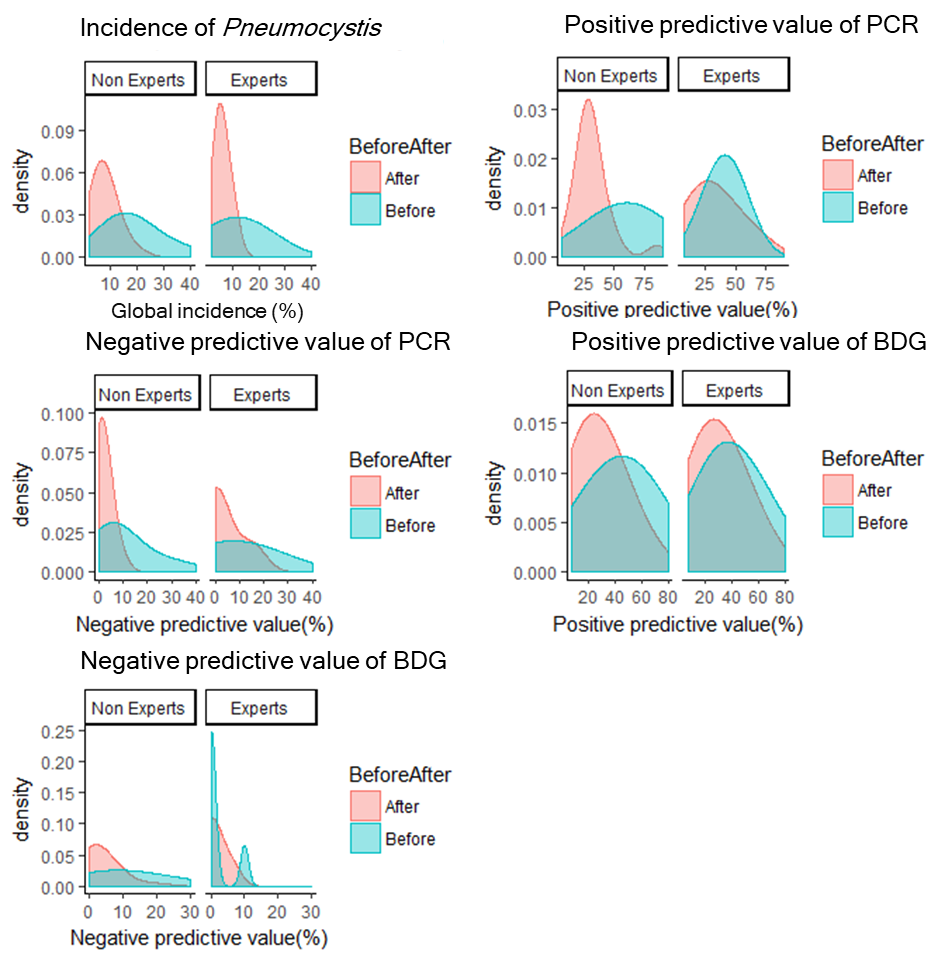


**Figure S9.** Pre/post evaluation of expert and non-expert physicians’ priors as regard to incidence of pneumocystis pneumonia and predictive value of PCR and BD glucan test in the high risk subgroup of critically ill immunocompromised patients with acute respiratory failure (i.e. with ground glass opacities, with lymphoid malignancy or HSCT and w/o prophylaxis).


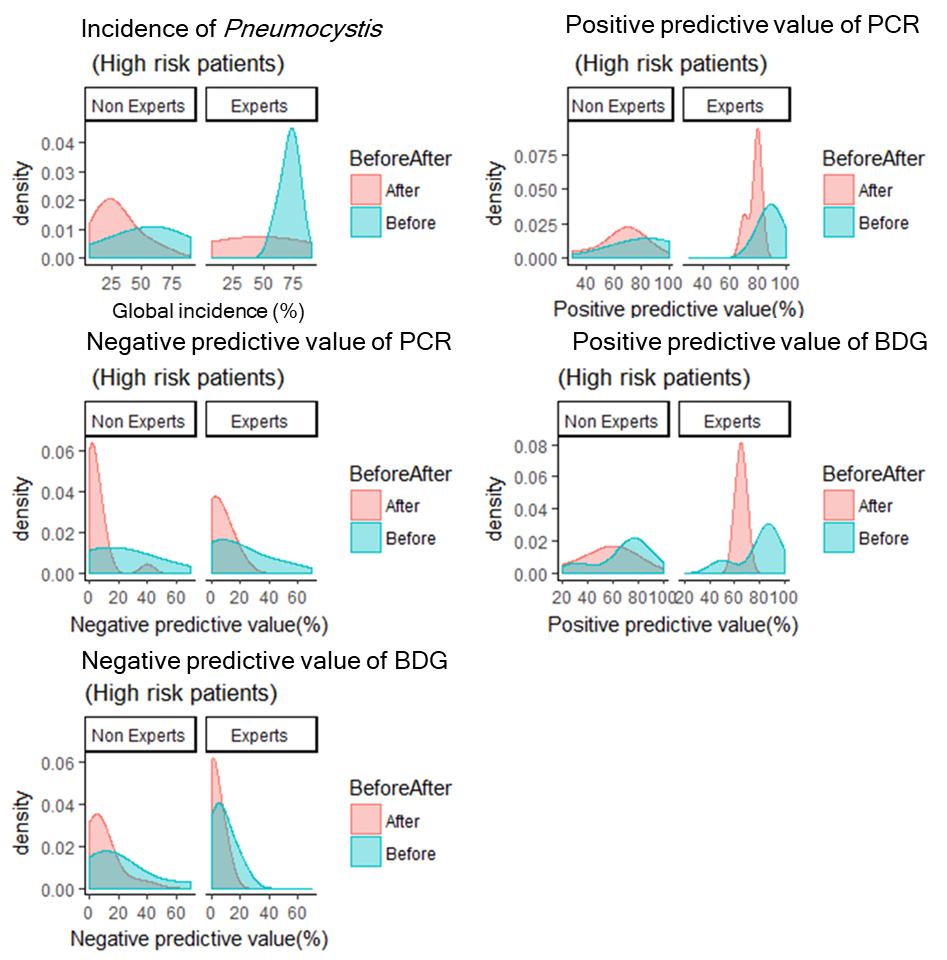
**Figure S10.** Example of Fagan’s nomogram with usual pre-test probability range (here 4.1%; 95% 3.8-4.4) highlighted and range of post-test probability assuming intermediate PCR test performance (Sensitivity 97.8%, Specificity 91.5%).
